# The Effects of Patients’ and Caregivers’ Characteristics on the Burden of Families Caring for Stroke Survivors

**DOI:** 10.3390/ijerph18147298

**Published:** 2021-07-08

**Authors:** Anna Kavga, Ioannis Kalemikerakis, Anastasios Faros, Maria Milaka, Dimitra Tsekoura, Maria Skoulatou, Ioanna Tsatsou, Ourania Govina

**Affiliations:** 1 Nursing Department, University of West Attica, 12243 Egaleo, Greece; akavga@uniwa.gr (A.K.); ikalemik@uniwa.gr (I.K.); kn18012@uniwa.gr (A.F.); ugovina@uniwa.gr (O.G.); 2 Nursing Department, 2nd Health Center of Athens, 12131 Athens, Greece; milakamaria86@gmail.com; 3 Hellenic Red Cross, 10672 Athens, Greece; tse.dimi@yahoo.com; 4 Nursing Department, General Hospital-Health Center of Naxos, 84300 Naxos, Greece; marianurseky@yahoo.gr; 5 Oncology-Hematology Department, Hellenic Airforce General Hospital, 11523 Athens, Greece

**Keywords:** stroke, family caregiver, depression, social support, burden

## Abstract

Background: Vascular strokes are the leading cause of long-term disability for adults. They impose high levels of burden on the patient, the family, and national healthcare systems worldwide. This study aimed to assess the effects of patients’ and caregivers’ characteristics on the perceptions of burden in families caring for a loved one living with stroke in Greece. Methods: Using purposive sampling, 109 dyads of patients and their respective caregivers were recruited from the Attica region. Patients completed a questionnaire that included personal characteristics and the Barthel Index, while caregivers completed a set of questionnaires—personal characteristics, revised Bakas Caregiving Outcomes Scale (BCOS), Personal Resource Questionnaire (PRQ 2000), and Center for Epidemiological Studies-Depression (CES-D). Results: Caregiving burden was linked to both patients’ and caregivers’ characteristics. A patient’s educational level, the number of family members living in the same house, the existence of equipment and facilities in the house, and the duration of provided care were associated with perception of greater burden. Regarding caregivers’ characteristics, those in good health had a significantly lower perception of burden. Higher PRQ 2000 scores were significantly associated with higher BCOS scores (less burden), and higher CES-D scores were significantly associated with lower BCOS scores (more burden). Conclusion: Caring for a loved one affected by stroke places a considerable burden on the caregiver. Systematic assessment and intervention strategies can help to identify caregivers at risk so that suitably targeted assistance may be provided.

## 1. Introduction

Vascular stroke is a major public health problem nationally and globally, with high morbidity and mortality, and a high recurrence rate that is associated with patients’ health behaviors [1]. However, in high-income countries, a substantial decrease in stroke incidence, mortality, and disability-adjusted life-years has been achieved during the last decade, most probably as the result of improvements in primary and secondary prevention, as well as acute stroke treatment and neurorehabilitation [2]. Nevertheless, stroke remains the leading cause of long-term disability, and stroke patients are the most frequent users of healthcare services [3].

With the increase in life expectancy in developed countries and the consequent upward shift in age distribution, stroke continues to be a major concern, with financial and other consequences for healthcare and social systems around the world. The majority of stroke survivors are discharged from hospital and are supported by their family, usually the spouse, who plays an important role in their rehabilitation, care, and emotional support [4,5]. Stroke survivors’ care is a multidimensional activity, the goals of which are constantly changing and adapting to the needs of the surviving patient [5]. The family becomes the informal caregiver, with family members caring for their relatives at a significant cost to themselves—this represents a valuable resource for the healthcare system and society [6].

Home care has been recognized as having a stressful impact on the caregiver’s health and quality of life [7]. Caregivers often feel unprepared for their new role, and this may result in distress and poor physical, mental, and social health [8]. These negative effects of care are described as burden, tension, and stress [9]. The caregiver burden refers to the negative emotions and tension experienced by the caregiver as a result of caring for patients with stroke or other chronic conditions [10]. It is a negative result of the care experience, which is exacerbated by the multiple roles and responsibilities that the caregiver undertakes [11]. The sudden change in life conditions following a stroke does not give the family time to adjust to the role of caregiver and may cause high levels of anxiety and depression that have a negative impact on the caregiver’s health and wellbeing [12]. Moreover, the caregiver’s health is linked to the patient’s physical, mental, psychological, and functional condition. Depending on the degree of patient dependency, the possibility of the caregiver experiencing burden, depression, exhaustion, and generally poor health increases [7]. A lack of social support will also increase the caregiver’s burden, making them the “second patient” in the family [13]. Caregivers who report a low burden have a better quality of life, while caregivers who report high levels of caregiving stress report poor physical health [14].

Although ample research into caregivers’ burden has been carried out, relatively few studies have addressed caregivers of stroke survivors in particular. Various tools have been constructed to measure the burden of caregivers of stroke patients, some of which measure the subjective burden, others the objective, and others the total caregiver burden. Almost all these scales take account of the different dimensions of burden: adequacy, negative emotions, social relationships, physical and mental stress, and financial problems [15].

The Greek healthcare system pushes the chronically ill out into society and home care, in most cases without the health services providing the required support for informal caregivers. The family must therefore take on the care of stroke patients almost entirely, relying upon the strong familial bonds that provide the motivation to care for a loved one. This provides a valuable service to the healthcare system, by reducing the cost of hospital or institutional care. In Greece, studies in the field of informal caregiving are scarce, especially in the case of stroke patients.

The aim of this study was to investigate the effects of patients’ and caregivers’ characteristics on the perceptions of burden in families caring for a loved one living with stroke in Greece.

## 2. Materials and Methods

### 2.1. Research Design

This was a cross-sectional, correlational study based on a purposive sampling of dyads that consisted of stroke survivors and their primary family caregivers.

### 2.2. Participants and Setting

The study was conducted during home visits within the community, in the wider region of Attica. The study’s population sample consisted of 109 stroke patients who had functional problems, and their 109 primary caregivers, who were family members.

The selection criteria for the patient sample included the inability to perform basic or complex functional activities of daily living as a result of the stroke, and the passage of 4 months since the stroke occurred. For caregivers, the inclusion criteria were to be a family member, to have the main responsibility for patient care, and to be living with the patient. People who did not speak Greek well and people who had mental or psychiatric problems were excluded from the study. If the stroke had left the patient unable to communicate, the caregiver provided the necessary information.

The collection of the sample took 13 months (from December 2017 to December 2018) and was performed during home visits (each lasting approximately 1 h), with the collaboration of neurologists and physicians from the private sector, the Hellenic Red Cross Home Care Service, and the National Rehabilitation Center.

### 2.3. Ethics

During home visits, the patient and the caregiver were verbally informed about the study, including its purpose, confidentiality, anonymity, voluntary participation, and the possibility of leaving the study at any time. Those who agreed to participate then signed a consent form. Permissions were obtained from the Ethics Committee of the Nursing Department of The National and Kapodistrian University of Athens, the Nursing Department of the Hellenic Red Cross, and the Board of Directors of the National Rehabilitation Center.

### 2.4. Instrumentation

The set of questionnaires addressed the patients’ and caregivers’ demographic and clinical characteristics, the patient’s functionality (Barthel Index) [16], the caregiving outcome (revised Bakas Caregiving Outcomes Scale) [17,18], the caregiver’s mental state (Center for Epidemiological Studies-Depression, CES-D) [19] and finally, the level of social support (Personal Resource Questionnaire, PRQ 2000) [20].

#### 2.4.1. Barthel Index

The Barthel Index (BI) was used to assess the patients’ functional capacity. This scale focuses on a patient’s ability to carry out basic activities of daily life at home. The original 10-question scale was used [16]. The Cronbach’s alpha coefficient was very high in this study (0.95).

#### 2.4.2. Revised Bakas Caregiving Outcomes Scale (BCOS)

The revised Greek BCOS is a 15-item questionnaire that assesses caregivers’ perceptions of changes in their lives as a result of providing care for the patient [18]. The 15 BCOS items measure changes in physical health, social functionality, and a caregiver’s subjective wellbeing on a 7-point scale. Grades 1, 2, and 3 represent a small, moderate, or large change for the worse, grade 4 means no change, and grades 5, 6, and 7 represent a small, moderate, or large change for the better. The range of total scores is thus from 15 to 105. An overall grade <40 means that caregivers believe their lives have changed noticeably for the worse. The Cronbach’s alpha coefficient for the total Greek BCOS score in the present study was 0.87.

#### 2.4.3. Center for Epidemiological Studies-Depression (CES-D)

The CES-D scale is targeted at symptoms of depression in a non-psychiatric population, such as the caregivers of stroke patients. It is a self-reported questionnaire, and its Greek version has satisfactory validity and reliability [21,22]. It includes twenty questions and six subscales that address physical and mental symptoms indicative of depression. Answers cover a 4-point scale (from 0 = rarely/never, to 3 = most of the time). The total score ranges from 0 to 60 and the cutoff point separating normal from pathologic is 16 [19]. A higher score denotes a higher level of depression [22]. In this study, the Cronbach’s alpha reliability coefficient was satisfactory (0.84).

#### 2.4.4. Personal Resource Questionnaire (PRQ 2000)

The 15-question PRQ 2000 [20] is designed to measure the perceived level of social support. It is graded on a 7-point Likert scale that corresponds to the level of agreement (from 1 = strongly disagree to 7 = strongly agree). The score range is from 15 to 105, where a higher score denotes a higher level of perceived social support. Cronbach’s alpha was calculated for this study at 0.84, which was considered satisfactory.

### 2.5. Statistical Analysis

Quantitative variables were expressed as mean values (standard deviation (SD)) or as median (interquartile range), while qualitative variables were expressed as absolute and relative frequencies. Student’s *t* tests and analysis of variance (ANOVA) were computed for the comparison of mean values. Pearson or Spearman correlation coefficients were used to explore the association of two continuous variables. Correlation coefficients between 0.1 and 0.3 were considered low, between 0.31 and 0.5 were considered moderate, and those over 0.5 were considered high. Multiple linear regression analysis was used with the BCOS scale as the dependent variable. The regression equation included terms for patients’ characteristics and caregivers’ characteristics, as well as the Barthel Index, CES-D and PRQ 2000 scores. Adjusted regression coefficients (β) with standard errors were computed from the results of the linear regression analyses. All reported *p*-values are two-tailed. With a sample size of 100 participants or more, it was calculated that the study would have more than 90% power to identify significant effects in a multiple regression model with 10 independent variables and at a significance level of 5%. Statistical significance was set at *p* < 0.05, and analyses were conducted using IBM SPSS Statistics for Windows, Version 22.0. Armonk, NY, USA: IBM Corp.

## 3. Results

The sample consisted of 109 patients and their caregivers, with mean age 69.3 years (SD 13.7 years) and 58.0 years (SD 13.5 years), respectively. Patients’ and caregivers’ characteristics are presented in Table 1.

Descriptions of all scales under study are presented in Table 2. The Greek BCOS score ranged from 20 to 86, with mean 48.3 (SD 13.3).

BCOS was significantly associated with patients’ educational level, with middle/high-school graduates or university alumni having lower values (Table 3). The more family members living in the same house, the lower the BCOS score. Moreover, in cases where there were equipment and facilities in the house, the BCOS score was significantly lower. The BCOS score was significantly lower as the number of months a patient was in need of care increased. As far as caregivers’ characteristics are concerned, those in good health had significantly higher BCOS scores. In addition, more months taking care of the patient and more daily hours of care were significantly associated with lower BCOS scores.

The BI was not significantly associated with BCOS (Table 4). In contrast, CES-D and PRQ 2000 were significantly associated with BCOS, in a negative and positive manner, respectively.

When multiple regression analysis was conducted, it was found that when patients were middle/high school graduates or university alumni, the BCOS score was significantly lower (higher burden) (Table 5). In addition, the BCOS score was significantly higher (lower burden) when the caregiver was in good health. More months of taking care of the patient, more daily hours of care, and more symptoms of depression were significantly associated with a lower BCOS score (higher burden). On the other hand, a higher PRQ 2000 score was significantly associated with a higher BCOS score (lower burden).

## 4. Discussion

This study evaluated the factors that influence a caregiver’s burden in caring for a patient after a stroke. The main findings were that a patient’s higher educational level, many family members living in the same house, the presence of equipment and facilities in the house (probably because of the patient’s greater disability), and the number of months the patient was in need of care significantly increased caregiver burden, lowering the BCOS score. The caregivers’ perception of burden was lower (higher BCOS score) for those caregivers in good health but higher (lower BCOS score) for those who provided many months and daily hours of care.

Factors such as the patient’s functional capacity (BI), socioeconomic class, and patient’s gender were not found to be significant in our study. However, Oni et al. [23], in their study in Nigeria, found that caregivers’ burden was significantly negatively affected by BI. In the same study, the burden was found to increase with the patient’s level of disability and the caregiver’s change of occupation but was inversely related to the time from the stroke event, the caregiver’s socioeconomic class, and their level of education, whereas we did not find either the caregiver’s socioeconomic level or the time elapsed since the stroke to be of significance.

In India, caregivers’ stress was affected primarily by the patient’s neurological status being moderately to severely affected, and by the patient being female [24]. The caregiver’s factors that induced stress were many caregiving hours (as in our study), anxiety, disturbed-night sleep, financial issues, younger age, and being a daughter-in-law.

In China, Zhu and Jiang [25] assessed 202 stroke survivor/caregiver dyads and concluded that physical function (BI), survivors’ depression and self-rated health, caregivers’ education level and age, attitude to caregiving, and satisfaction with caregiving showed an association with caregiver burden.

In addition, a stroke survivor’s functional disability and the gender of an informal caregiver were associated with informal caregiver burden in the USA [26], though we found no such associations in our study. Pucciarelli et al. [27] performed a 12-month longitudinal study in order to detect the changes in caregivers’ quality of life, anxiety, depression, and burden, and their predictors. A higher burden was predicted by the caregiver being male, the caregiver not living with the patient, and the patient’s poorer physical capacity (lower BI).

Partners (*n* = 183) of stroke patients in the Netherlands experienced a high care burden, anxiety, and depressive symptoms that were associated with partner variables (younger age, relationship satisfaction, proactive coping, self-efficacy, everyday social support, burden, anxiety, and depressive symptoms) and patient variables (stroke severity and depressive symptoms) [28]. Patients’ age, comorbidities, and cognitive function, as well as the gender and self-reported health of caregivers, were correlated with caregiver burden at 6 months following stroke [29]. In a study by Olai et al. [30] in Sweden, caregiver burden increased with low informal caregiver support, patient’s age, and with low functional capacity/BI. Caregiver health, patient’s gender, and time spent caregiving were also related to the burden in Poland [31]. We did not find associations between caregiver burden and patient’s or caregiver’s age and gender, nor with patient’s functionality level/BI. However, we did find that a good awareness of personal health was associated with a smaller burden. A recent meta-analysis concluded that patients’ better capability for daily living and psychological health reduced caregiver burden [32]. The caregiving workload was positively correlated with the caregiver burden, suggesting that the more tasks the caregivers undertook, the more burden they experienced. This may explain our finding that the caregiver burden was greater in households with many members, where those remaining at home would be obliged to take on more duties. A recent Greek study found that caregivers downplay the importance of their personal health when taking on intensive care roles [33], further exacerbating the burden they experience.

We also found a correlation between caregivers’ burden and depression. This finding is in line with those of previous researchers [23,26,28,31]. In particular, the severity of the caregivers’ burden correlated positively with the severity of depression, and higher depressive symptoms were related to life changes for the worse (higher burden) [34]. Depression and anxiety of caregivers were also strong predictors of worsened caregiver burden in a recent meta-analytic study [32].

Furthermore, we found a significant correlation between social support and caregivers’ burden score. Social support is seen to be beneficial for caregivers’ health [35]. Chung et al. [36], in South Korea, reported that persistent depressive symptoms were linked to the highest level of burden, and the lowest level of family function and perceived availability of social support, at both assessment times. Moreover, a low level of municipal social service support was associated with a significantly greater caregiver burden [30], while the burden was less in caregivers who received a high level of social support [31].

It is notable that in all studies, including the present one, the typical primary caregiver was female (spouse/partner/daughter) [23,24,27,28,29,30,31,34,35,36]. Women are the primary caregivers in the family setting. They seem to see it as a moral obligation and are resigned to being assigned the role of caregiver, even if they do not recognize this themselves. Thus, female caregivers whose physical and psychological health is affected experience higher levels of care burden and lower levels of social support [35].

In general, the results of different studies appear to indicate that the factors affecting caregiver burden differ from country to country. This may be due to cultural differences that affect the perception and the whole experience of burden and its outcomes. These differences have been acknowledged in terms of caregiver burden and distress, attitudes and norms, appraisal, coping, help seeking, and social support among caregivers belonging to various ethnic and cultural groups [37].

The limitations of this study included the relatively small sample of patient/caregiver dyads and the convenience sampling, making it difficult to generalize the results to all caregivers of patients affected by stroke. Moreover, the cross-sectional design does not allow us to draw firm conclusions about the relationship between patient/caregiver characteristics and burden, especially in the long term. Future research could focus on prospective studies and on monitoring the same dyads (caregiver/patient) at different time intervals during care. Anyone who did not speak Greek well was excluded from the study. Although we recognize the fact that people not speaking the local language are often those who encounter the most difficulties coping with stroke, the necessary resources, in the form of translators for other languages, were not available to us. Finally, the questionnaires used to collect the data have the limitations of all subjective assessments. A qualitative study might be able to reveal the impact of the burden on caregivers and the relative factors with more accuracy.

## 5. Conclusions

The present study investigated the effects of patients’ and caregivers’ characteristics on the burden of families that care for patients with stroke. A higher caregiver burden was associated with depressive mood, social support, patient’s educational level, living conditions, available equipment and facilities, and caregiving duration, while a lower burden was related to the caregiver’s good health.

These findings are of major importance, since the present study is one of the few conducted in the Greek population and may increase nurses’ awareness of caregivers’ burden and needs during the care of a person affected by a chronic and debilitating condition. Early detection and identification of caregivers who are at risk of physical and psychological problems in the early stages of caregiving could lead to appropriate burden-relieving strategies and interventions.

A deeper understanding of the concept of burden and its impact on the physical and psychological health of the whole caregiving family could add another dimension to the role of the nurse in community nursing and in promoting family health. Furthermore, this study could act as a trigger for more research about informal care and its impact on the health of caregivers, in order to highlight the multiple needs of caregivers and at the same time to illuminate the need for and importance of community and family nursing.

## Figures and Tables

**Table 1:** Sample characteristics.

	*N* (%)
Patients	
Gender	
Males	56 (51.4)
Females	53 (48.6)
Age, mean (SD)	69.3 (13.7)
Educational level	
Primary school at most	65 (59.7)
Middle/high school or university	44 (40.3)
Married	72 (66.1)
Number of children, mean (SD)	2.2 (1.3)
Family members living in the same house, mean (SD)	2.8 (1.1)
Annual family income	
<10,000€	62 (56.8)
>10,000€	47 (43.2)
Equipment and facilities in the house	39 (35.8)
Diagnosis	
Right hemiplegia	52(47.7)
Left hemiplegia	57 (52.3)
Months in need of care, median (IQR)	10 (5–36)
Caregivers	
Gender	
Males	35 (32.1)
Females	74 (67.9)
Age, mean (SD)	58.0 (13.5)
Relation with patient	
Spouse	55 (50.5)
Son/daughter	38 (34.9)
Other	16 (14.6)
Educational level	
Primary school at most	38 (34.9)
Middle/high school or university	71 (65.1)
Married	83 (76.1)
Number of children, mean (SD)	1.7 (1.1)
Working status	
Employed	33 (30.3)
Pensioner	58 (53.2)
Other	18 (16.5)
Annual family income	
<10,000€	59 (54.2)
>10,000€	50 (45.8)
Daily hours of care, mean (SD)	13.2 (6.4)
Health condition	
Good	44 (40.4)
Moderate	54 (49.5)
Poor	11 (10.1)

SD: standard deviation. IQR: Interquartile range.

**Table 2:** Descriptions of study scales.

	Minimum	Maximum	Mean	SD
Greek BCOS	20	86	48.3	13.3
Barthel Index	0	95	44.0	30.6
CES-D	5	47	21.7	10.1
PRQ 2000	43	102	77.7	13.8

SD: standard deviation.

**Table 3:** Univariate analysis for Greek BCOS with patients’ and caregivers’ characteristics.

	Greek BCOS	
	Mean (SD)	*p*
Patients		
Gender		
Males	46.3 (13.8)	0.116 ^+^
Females	50.4 (12.6)	
Age, r ^1^	−0.03	0.762
Educational level		
Primary school at most	50.7 (12.6)	0.024 ^+^
Middle/high school or university	44.8 (13.8)	
Married		
No	48.4 (12.3)	0.938 ^+^
Yes	48.2 (14.0)	
Number of children, r ^1^	0.01	0.885
Family members living in the same house, r ^1^	−0.25	0.008
Annual family income		
<EUR 10,000	46.3 (13.0)	0.067 ^+^
>EUR 10,000	51.0 (13.4)	
Equipment and facilities in the house		
Yes	43.9 (12.3)	0.010 ^+^
No	50.7 (13.4)	
Diagnosis		
Right hemiplegia	49.9 (13.6)	0.163 ^+^
Left hemiplegia	48.0 (13.1)	
Months in need of care, r ^2^	−0.34	<0.001
Caregivers		
Gender		
Males	49.9 (14.3)	0.394 ^+^
Females	47.5 (12.9)	
Age, r ^1^	−0.10	0.304
Relation with patient		
Spouse	48.5 (14.7)	0.843 ^++^
Son/daughter	47.4 (12.6)	
Other	49.6 (10.6)	
Educational level		
Primary school at most	50.4 (14.7)	0.237 ^+^
Middle/high school or university	47.2 (12.5)	
Married		
No	47.7 (11.7)	0.781 ^+^
Yes	48.5 (13.9)	
Number of children, r ^1^	−0.06	0.504
Working status		
Employed	51.2 (12.1)	0.293 ^++^
Pensioner	47.4 (14.0)	
Other	45.8 (13.0)	
Annual family income		
<EUR 10,000	47.0 (13.5)	0.267 ^+^
>EUR 10,000	49.8 (13.1)	
Health condition		
Poor/moderate	44.7 (13.2)	<0.001 ^+^
Good	53.7 (11.7)	
Months taking care of the patient, r ^2^	−0.32	0.001
Daily hours of care, r ^1^	−0.40	<0.001

^+^ Student’s *t* test; ^++^ ANOVA; ^1^ Pearson’s correlation coefficient; ^2^ Spearman’s correlation coefficient.

**Table 4:** Pearson’s correlation coefficients between Greek BCOS Barthel Index, CES-D, and PRQ 2000.

	Greek BCOS
Barthel Index	0.15
CES-D	−0.36 **
PRQ 2000	0.29 *

* *p* < 0.01; ** *p* < 0.001.

**Table 5:** Multiple linear regression analysis results with Greek BCOS as dependent variable, patients’ and caregivers’ characteristics, Barthel Index, CES-D, and PRQ 2000 as independent variables, using stepwise method.

	β ^+^	SE ^++^	*p*
Patient’s Educational level			
Primary school at most (reference)			
Middle/high school or university	−6.27	2.09	0.003
Caregiver’s health condition			
Poor/moderate (reference)			
Good	6.33	2.23	0.005
Months taking care of the patient	−0.14	0.04	0.002
Daily hours of care	−0.36	0.18	0.047
CES-D	−0.25	0.11	0.024
PRQ 2000	0.19	0.08	0.012

^+^ regression coefficient; ^++^ standard error.

## Data Availability

The original data presented in this study are available by request to the authors.
